# Borneol Alleviates Polyethylene Microsphere-Induced Cerebral Microcirculatory Dysfunction with Reduced NET-Related Markers

**DOI:** 10.3390/brainsci16070701

**Published:** 2026-06-30

**Authors:** Yanlong Zhou, Dongdong Jia, Wei Hou, Zengcai Liu, Yinju Liu, Lanying Chen, Ronghua Liu

**Affiliations:** 1 National Pharmaceutical Engineering Center for Solid Preparation of Chinese Herbal Medicine, Jiangxi University of Chinese Medicine, Nanchang 330006, China; 2 Jiangxi Provincial Key Laboratory of Effective Material Basis of TCM, Jiangxi University of Chinese Medicine, Nanchang 330004, China; 3 International Education College, Jiangxi University of Chinese Medicine, Nanchang 330006, China; 4 School of Pharmacy, Jiangxi University of Chinese Medicine, Nanchang 330004, China; 19890068@jxutcm.edu.cn

**Keywords:** borneol, neutrophil extracellular traps, microcirculation, cerebral microvascular perfusion, brain injury, thromboinflammation, oxidative stress

## Abstract

**Highlights:**

**What are the main findings?**

- Borneol attenuated PMA-induced NET formation in rat bone marrow-derived neutrophils.
- Borneol improved neurological function, cerebral microvascular perfusion, hemorheology, and inflammatory/oxidative markers in a microsphere-induced rat model.

**What are the implications of the main findings?**

- NET-related markers were elevated in microcirculatory dysfunction-associated brain injury.
- Borneol may provide preliminary neurovascular protection in microcirculatory dysfunction-related brain injury, an effect that is accompanied by reductions in NET-related markers, although the causal relationship between these observations remains to be established.

**Abstract:**

Background/Objectives: Neutrophil extracellular traps (NETs) contribute to thromboinflammation and microvascular obstruction after ischemic brain injury. Borneol has anti-inflammatory and microcirculation-related pharmacological activities, but its effects on cerebral microcirculatory dysfunction and NET-related changes remain unclear. This study aimed to determine whether borneol attenuates polyethylene microsphere-induced cerebral microcirculatory dysfunction and to examine its association with NET-related markers. Methods: Rat bone marrow-derived neutrophils were stimulated with PMA with or without borneol. Male Sprague–Dawley rats were subjected to polyethylene microsphere-induced cerebral microcirculatory dysfunction and treated intragastrically with borneol (0.1, 0.2, or 0.4 g/kg). NET formation, neurological deficits, hemodynamics, cerebral microvascular perfusion, hemorheology, histopathology, inflammatory and oxidative stress markers, and NET-related molecules were assessed by immunofluorescence, ELISA, quantitative PCR, and Western blotting. Results: Borneol reduced PMA-induced NET formation in vitro. In vivo, borneol improved neurological scores, hemodynamic indices, microvascular perfusion, hemorheological parameters, and histopathological injury. It also reduced serum TNF-α, IL-1β, and ROS, decreased cerebral MPO/CitH3 signals, and downregulated MPO, *PADI4*/PAD4, CitH3, TNF-α, IL-6, IL-8, ICAM-1, and MIP2. Conclusions: These findings suggest that borneol alleviates polyethylene microsphere-induced cerebral microcirculatory dysfunction, accompanied by reductions in NET-related markers. However, as borneol was administered prophylactically before model induction, these results should be interpreted with caution and do not directly support a post-insult therapeutic application.

## 1. Introduction

Microcirculatory dysfunction is characterized by impaired capillary perfusion, increased microvascular resistance, abnormal hemodynamics, and insufficient tissue oxygen supply. In brain tissue, disorders of microcirculation can lead to persistent hypoperfusion and subsequent tissue injury, even when major vessels remain unobstructed. Although microcirculatory impairment is clinically relevant to acute ischemic stroke and post-recanalization perfusion failure, the present study focused on polyethylene microsphere-induced cerebral microcirculatory dysfunction as an experimental model of mechanical microvascular obstruction [1,2,3,4].

The mechanisms underlying cerebral microcirculatory dysfunction are multifactorial, involving intravascular, vascular wall, and extravascular factors. Contributing elements such as microthrombi, microemboli, adhesion and aggregation of blood components, neutrophil retention, endothelial swelling, and vascular dysfunction may all impair microvascular perfusion [4,5,6]. Among these factors, neutrophil recruitment and neutrophil extracellular traps (NETs) have garnered increasing attention. NETs are extracellular chromatin-based structures that are decorated with granular proteins, including myeloperoxidase (MPO) and neutrophil elastase (NE) [7]. Excessive formation of NET may promote microvascular obstruction, disrupt the blood–brain barrier, contribute to thrombosis, and amplify inflammation following ischemic or microvascular injury [8,9,10].

NET formation is closely associated with the reactive oxygen species (ROS)-dependent activation of peptidylarginine deiminase 4 (PAD4), which promotes histone H3 citrullination, chromatin decondensation, and the extracellular release of NET-related components [7,11]. This ROS/PAD4/citrullinated histone H3 (CitH3) axis represents a critical juncture at which pharmacological interventions may modulate NETosis. Previous studies have demonstrated that NET-related markers are elevated in experimental ischemic brain injury, and that NET-targeting strategies, including DNase I, PAD4 inhibition, and neutrophil depletion, can reduce microvascular obstruction and enhance neurological outcomes in selected models [8,12,13]. These findings indicate that NET-related inflammatory responses may contribute to brain injury associated with impaired microvascular perfusion.

Borneol (BO) is a traditional aromatic resuscitative medicine known for its reported pharmacological effects, including anti-inflammatory, antioxidant, blood–brain barrier protection, and enhancement of microcirculation [14]. Previous studies have demonstrated that borneol can mitigate oxidative stress and inflammatory responses associated with cerebral ischemia–reperfusion injury [15,16]. Notably, the antioxidant activity of borneol may directly influence the initiation of NETosis; the respiratory burst in activated neutrophils serves as a primary source of reactive oxygen species (ROS) that drive PAD4 activation and subsequent histone citrullination. Borneol-mediated ROS scavenging could theoretically limit this cascade. Furthermore, borneol has been shown to inhibit NF-κB signaling and the production of downstream inflammatory cytokines [15], which may indirectly diminish the pro-inflammatory environment that promotes NET formation. However, it remains unclear whether borneol influences cerebral microcirculatory dysfunction-related brain injury are paralleled by changes in NET-related inflammatory pathways, beyond its general anti-inflammatory effects. Consequently, this study aims to investigate the protective effects of borneol against microcirculatory dysfunction-related brain injury and to explore whether these effects are associated with reductions in NET-related markers.

This study first examined the effect of borneol on PMA-induced NET formation in rat bone marrow-derived neutrophils in vitro. Subsequently, a rat model of polyethylene microsphere-induced cerebral microcirculatory dysfunction was established. This model induces multifocal mechanical microvascular obstruction through inert microspheres, resulting in hypoperfusion, increased microvascular resistance, and secondary neuroinflammatory responses. These pathological features partially replicate selected aspects of no-reflow-like microvascular perfusion impairment, providing a valuable experimental setting to evaluate the effects of borneol on microsphere-induced microvascular obstruction, impaired cerebral perfusion, and associated changes in NET-related markers. In contrast to transient MCAO or photothrombotic models [17,18], the microsphere model emphasizes microcirculatory dysfunction without large-vessel occlusion or direct photochemical endothelial injury. However, it does not completely reproduce thrombotic occlusion, reperfusion dynamics, or the clinical phenomenon of post-recanalization no-reflow.

## 2. Materials and Methods

### 2.1. Reagents

Rat bone marrow neutrophil isolation kit and red blood cell lysis buffer were purchased from Solarbio (Beijing, China). RPMI-1640 medium was obtained from Thermo Fisher Scientific (Waltham, MA, USA). Polyethylene microspheres were purchased from Yiyuan Biotechnology (Guangzhou, China). DiIC12 was obtained from Macklin Biochemical Co., Ltd. (Shanghai, China), and DiIC18 was purchased from Merck Chemical Technology (Shanghai, China). OCT embedding compound was obtained from Sakura Finetek (Torrance, CA, USA). Rat reactive oxygen species (ROS), interleukin-1β (IL-1β), and tumor necrosis factor-α (TNF-α) ELISA kits (lot numbers: MM-21264R1, MM-0047R1, and MM-0180R1, respectively) were purchased from Jiangsu Meimian Biotechnology Co., Ltd. (Yancheng, China). Total RNA extraction kit (catalog number: RN7101) was obtained from Aidlab Biotechnologies Co., Ltd. (Beijing, China). The 2 × Dual SYBR Green qPCR Mix (Universal ROX) (catalog number: PC6202) and TRUEscript RT MasterMix (OneStep gDNA Removal) reverse transcription kit (catalog number: PC7002) were purchased from HuaXuan Biotechnologies Co., Ltd. (Nanchang, China). The BCA protein assay kit (lot number: 3871400) was purchased from Yeasen Biotechnology (Shanghai, China). Anti-MPO, anti-PAD4, and anti-GAPDH antibodies were purchased from Proteintech (Wuhan, China) (catalog numbers: CL488-66177, 17373-1-AP, and 60004-1-Ig, respectively). Anti-CitH3 and anti-TNF-α antibodies were obtained from Abcam (Cambridge, UK) (catalog numbers: ab281584 and ab307164, respectively). Anti-rabbit and anti-mouse secondary antibodies were purchased from HuaAn Biotechnology Co., Ltd. (Hangzhou, China) (lot numbers: HA1121 and HO1117, respectively). YF 647 Goat Anti-Rabbit IgG was purchased from Suzhou Yooland Biotech Co., Ltd. (Suzhou, China) (lot number: Y6109L).

### 2.2. Animals

Male Sprague–Dawley rats (6 weeks old, 240 ± 20 g, SPF grade; *n* = 36) were obtained from Henan Skbex Biotechnology Co., Ltd. (Anyang, China) (License No. SCXK (2020-0005)). Animals were housed under standard laboratory conditions with a 12 h light/dark cycle at 24 ± 2 °C and 50% ± 10% relative humidity, with free access to food and water. After a 5-day acclimatization period, 36 rats were randomly assigned to the six experimental groups (*n* = 6 per group) for the in vivo study. All 36 animals survived to the designated endpoints without any mortality or unexpected events. No animals or samples were excluded from the final analysis. For molecular assays (qPCR, Western blotting, and immunofluorescence quantification) that required tissue processing, 3 animals per group were randomly selected from the 6 available animals as biological replicates, as specified in the respective method sections. All experimental procedures were approved by the Animal Ethics Committee of Jiangxi University of Chinese Medicine (Approval No. JZSYDWLL-20200801). All subsequent procedures and analyses were performed blinded to group assignment. No animals were excluded from the analysis.

### 2.3. Drug Preparation

Borneol (BO) was sourced from Jiangxi Linke Longnao Technology Co., Ltd. (Ji’an, China), batch No. 20230303, and contained 98.8% d-borneol, which complied with the specifications of the Chinese Pharmacopoeia (2020 edition). To ensure adequate bioavailability and uniformity for intragastric administration, the corresponding mass of borneol powder was accurately weighed. Sterile purified water was subsequently added to reach the final volume, and the mixture was homogenized using an ultrasonic instrument to prepare a uniform microemulsion suspension. The prepared drug solution was stored at 4 °C, protected from light and vortexed thoroughly before each administration. The positive control drug nicorandil was prepared in purified water.

### 2.4. Isolation of Bone Marrow-Derived Neutrophils and PMA-Induced NET Formation

**Figure A1:**
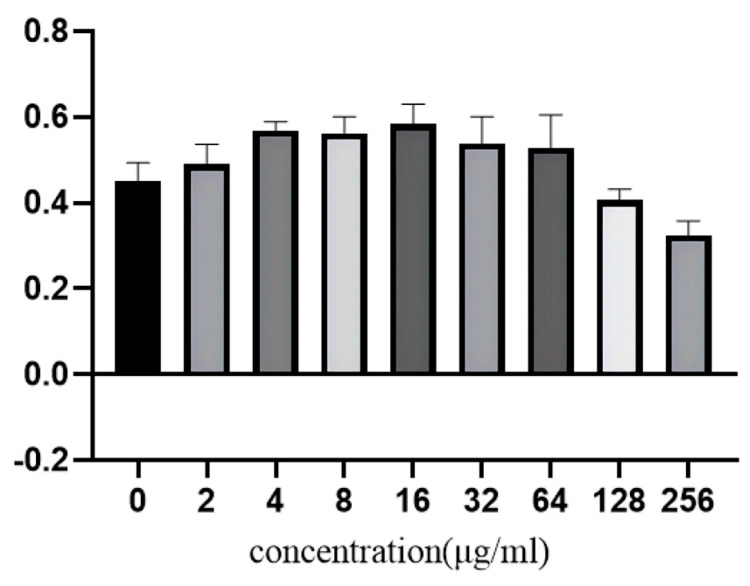
Effects of different concentrations of borneol on the viability of rat bone marrow-derived neutrophils. Cells were treated with borneol at concentrations of 0, 2, 4, 8, 16, 32, 64, 128, and 256 μg·mL^−1^, and cell viability was evaluated using the CCK-8 assay. Based on these results, 4, 8, and 16 μg·mL^−1^ borneol were selected as the low-, medium-, and high-dose treatment concentrations, respectively. Data are presented as mean ± SD.

Bone marrow was harvested from the tibias and femurs of normal rats, flushed with pre-cooled RPMI 1640 medium, and filtered to obtain a bone marrow cell suspension. The cells were resuspended in RPMI 1640 medium containing 5% fetal bovine serum (FBS). After counting, a portion of the cells was subjected to Wright–Giemsa staining to assess cell purity, and the remaining cells were seeded into 24-well plates at a density of 5 × 10^5^ cells/well. After incubation at 37 °C in a humidified atmosphere containing 5% CO_2_ for 1 h, the optimal drug concentration was determined by the CCK-8 assay. Based on the CCK-8 results (Figure A1), the cells were divided into the Sham group, Model group, nicorandil group (NIC, 32 μg·mL^−1^), and low-, medium-, and high-dose borneol groups (BO-L, 4 μg·mL^−1^; BO-M, 8 μg·mL^−1^; and BO-H, 16 μg·mL^−1^). Neutrophils were pretreated with borneol for 1 h prior to PMA stimulation. Except for the Sham group, all groups were stimulated with PMA (500 nmol·L^−1^) for 4 h to induce NET formation.

### 2.5. Immunofluorescence Staining of MPO and CitH3 in Neutrophils

After PMA stimulation, the cells were fixed with 500 μL of 4% paraformaldehyde for 15 min. Following PBS washing, the cells were permeabilized with 0.5% Triton X-100 for 5 min. After washing with PBS, the cells were incubated with blocking solution for 20 min and then gently washed twice. The cells were subsequently incubated with primary antibodies against MPO (1:200) and CitH3 (1:1800) at room temperature for 1 h, followed by incubation at 4 °C overnight. After three washes with PBS (5 min each), the cells were incubated with goat anti-rabbit Alexa Fluor 647-conjugated secondary antibody (1:200) and goat anti-mouse Alexa Fluor 488-conjugated secondary antibody (1:200) at room temperature for 2 h. The cells were then washed three times with PBS (5 min each), stained with DAPI at room temperature for 10 min, and washed twice with PBS (5 min each). Finally, 200 μL PBS was added to each well, and images were acquired using a laser scanning confocal microscope (Leica Microsystems, Wetzlar, Germany). The fluorescence signals of CitH3 and MPO were quantified using ImageJ software (version 1.53t).

### 2.6. Experimental Grouping and Model Establishment

After a 5-day acclimatization period, the 36 rats were randomly assigned into six groups (*n* = 6 per group): Sham group, Model group, nicorandil group (NIC, 1 mg·kg^−1^), low-dose borneol group (BO-L, 0.1 g·kg^−1^), medium-dose borneol group (BO-M, 0.2 g·kg^−1^), and high-dose borneol group (BO-H, 0.4 g·kg^−1^). Borneol (BO) (sourced from Jiangxi Linke Longnao Technology Co., Ltd., batch No. 20230303) contained 98.8% d-borneol. The doses of borneol were selected based on the recommended adult oral dose of natural borneol recorded in the Chinese Pharmacopoeia (2020 edition; 0.3–0.9 g/day) and converted according to body surface area normalization. Using the upper-end adult dose of 0.9 g/day and assuming a 60 kg adult body weight, the corresponding human dose is approximately 0.015 g/kg. Based on the commonly used human-to-rat conversion factor of 6.2, the rat equivalent dose was calculated as approximately 0.093 g/kg, which was rounded to 0.1 g/kg as the low dose. The medium and high doses were set as 0.2 and 0.4 g/kg, respectively, representing 2-fold and 4-fold multiples of the low dose to evaluate dose–response effects. Drugs were administered intragastrically once daily for 7 consecutive days. Rats in the Sham and Model groups received an equal volume of purified water.

On day 6, 45 min after administration, the model was established. Rats in the Sham group received left ventricular injection of PBS, whereas rats in the other groups received left ventricular injection of a polyethylene microsphere suspension. For modeling, rats were first anesthetized with urethane (1.0 g·kg^−1^, intraperitoneally), followed by inhalation of 5% isoflurane for 3 min. The depth of anesthesia was monitored by assessing the pedal withdrawal reflex and respiratory rate. After loss of the righting reflex, a needle was inserted at the point of maximal cardiac impulse between the fourth and fifth left intercostal spaces, and 0.5 mL of polyethylene microsphere suspension (42 μm, 60,000 particles) was slowly injected. Successful left ventricular injection was defined by correct needle placement at the point of maximal cardiac impulse, continuous blood reflux into the needle hub, smooth injection without obvious resistance, and subsequent confirmation of microsphere distribution in cerebral microvessels. At 24 h after modeling, the rats were given the final drug administration. Neurological behavioral evaluation, hemodynamic measurement, serum collection and brain tissue harvesting were sequentially conducted 45 min after this last treatment.

### 2.7. Neurological Deficit Assessment

Neurological deficits were evaluated 24 h after model establishment using the Longa scoring system in a double-blind manner: 0 points for no neurological deficits; 1 point for inability to fully extend the contralateral forelimb; 2 points for circling toward the contralateral side during walking; 3 points for tilting toward the contralateral side during standing or walking; 4 points for the inability to walk independently and loss of consciousness. A higher score indicates more severe neurological dysfunction.

### 2.8. Hemodynamic Measurement

After anesthesia, cardiovascular function-related parameters were recorded synchronously using a Power Lab electrophysiological instrument (ADInstruments Pty Ltd., Bella Vista, Australia): left ventricular pressure was recorded via a catheter inserted through the right common carotid artery, carotid blood flow was monitored with a left common carotid artery flow probe, and blood pressure was detected via femoral artery cannulation.

### 2.9. DiI Perfusion-Based Visualization of Cerebral Microvasculature

The rats were randomly selected in each group for tracheal intubation and assisted ventilation. After thoracotomy and exposure of the heart, the descending aorta was clamped. A 25-gauge butterfly needle was inserted into the left ventricle, perfusing PBS solution at a rate of 1–2 mL·min^−1^ for 3 min, followed by DiI C12/C18 mixed fluorescent dye at 3 mL·min^−1^ for approximately 5 mL. After waiting for 2.5 min, 10% formaldehyde solution was perfused at 1–2 mL·min^−1^ for about 4 min to achieve cerebrovascular labeling and in situ fixation. Brain tissue was fixed in 10% formaldehyde for 24 h, dehydrated in 30% sucrose solution for 12 h, embedded in OCT, and sectioned into 25 μm cryosections. Layer scanning imaging was performed under a laser confocal microscope (Leica, Berlin, Germany). Fields of view were randomly selected around polyethylene microspheres, and cerebrovascular morphology and functional parameters were quantitatively analyzed using AngioTool software (version 0.6a). The microvascular resistance was further estimated via the Hagen–Poiseuille equation R = 8 ηL/πr^4^, where R represents microvascular resistance, η denotes blood viscosity, L is vessel length, and r stands for vessel radius.

### 2.10. Hemorheological and Hematological Analyses

After hemodynamic monitoring, blood was immediately drawn from the abdominal aorta of the animal, and whole blood viscosity (WBV) was measured using a blood rheometer (China Southern CNC Co., Ltd., Chongqing, China) at shear rates of 1, 30, and 200 s^−1^. Heparinized blood was centrifuged, and the supernatant was collected to determine plasma viscosity (PV) and fibrinogen (FIB) levels. Additionally, heparinized whole blood was analyzed using an automated hematological analyzer to determine the following parameters: platelet count (PLT), neutrophil percentage (Neu%), mean platelet volume (MPV), and hematocrit (HCT).

### 2.11. Histopathological Examination

Fresh brain tissues were fixed in 10% formaldehyde for 24 h. Tissues were then dehydrated through a graded ethanol series (50%, 70%, 85%, 95%, and 100%), cleared in xylene, embedded in paraffin, and sectioned at 5 μm thickness. Sections were baked at 60 °C for 2 h, deparaffinized in xylene (2 × 10 min), and rehydrated through a descending graded ethanol series. Sections were stained with Harris hematoxylin for 8 min, rinsed in running water for 3 min to stop staining, differentiated in 1% HCl-ethanol for 5 s, and blued in running water for 5 min. Counterstaining was performed with eosin for 1 min, followed by dehydration through a graded ethanol series, clearing in xylene, and mounting with neutral resin. Histopathological changes in tissue architecture were observed under a light microscope (Nikon Corporation, Tokyo, Japan) at magnifications of ×20 and ×40. Histopathological injury was semi-quantitatively evaluated on a 0–4 scale according to the severity of neuronal damage, edema or vacuolization, hemorrhage or vascular extravasation, and inflammatory infiltration (0, no obvious injury; 1, mild injury; 2, moderate injury; 3, severe injury; 4, extensive injury). In addition, morphologically damaged neurons were identified based on cell shrinkage, eosinophilic cytoplasm, nuclear pyknosis, or loss of nuclear staining.

### 2.12. Immunofluorescence Staining of MPO and CitH3 in Brain Tissue

Brain tissues were fixed in 10% formaldehyde, dehydrated in 30% sucrose solution for 12 h, embedded in OCT compound, and sectioned into 5 μm-thick cryosections. Sections were washed with PBS for 5 min. After blocking with blocking buffer at room temperature for 1 h, sections were washed three times with PBS. Primary antibodies—anti-MPO (1:200) and anti-CitH3 (1:1800)—were applied and incubated overnight at 4 °C. Following three washes with PBS (5 min each), corresponding fluorescent secondary antibodies—goat anti-rabbit 647 (1:200) and goat anti-mouse 488 (1:200)—were added and incubated at room temperature for 1 h. Sections were then washed three times with PBS (5 min each), stained with DAPI for 10 min at room temperature, and washed again three times with PBS (5 min each). Fluorescence images were acquired using a laser confocal microscope (Leica, Germany). Representative line-scan fluorescence intensity profiles of MPO and CitH3 signals were generated using ImageJ software to illustrate their spatial distribution in brain tissue.

### 2.13. Determination of Serum TNF-α, IL-1β, and ROS

Blood was collected from the abdominal aorta using ordinary vacuum blood collection tubes and centrifuged at 3500 r·min^−1^ for 15 min at 4 °C to obtain serum. Serum TNF-α and IL-1β levels were measured using corresponding ELISA kits, and oxidative stress-related activity was assessed using a commercial ROS assay kit according to the manufacturers’ protocols. Briefly, standards and serum samples were added to the microplate wells, followed by the corresponding enzyme-linked reagents and chromogenic substrate. After termination of the reaction, the absorbance was measured, and concentrations or relative levels were calculated based on standard curves. Because ROS are highly labile, the ROS assay result was interpreted as an indirect indicator of systemic oxidative stress rather than a direct measurement or differentiation of individual unstable ROS, such as superoxide anion, hydrogen peroxide, or hydroxyl radicals.

### 2.14. Quantitative Real-Time PCR

Total RNA was extracted from brain tissue using a total RNA extraction kit, and cDNA was synthesized by reverse transcription using a reverse transcription kit. The qPCR reaction system was prepared according to the instructions of the 2 × Dual SYBR Green qPCR premix kit and amplified in a 96-well plate. The reaction conditions were pre-denaturation at 95 °C for 2 min, followed by 40 cycles of 95 °C for 5 s and 60 °C for 30 s. β-actin served as the internal control. The expression of each target gene mRNA was calculated using the 2^−ΔΔCt^ method. Primer sequences are listed in Table 1. For qPCR analysis, brain tissue samples from 3 randomly selected animals per group (selected from the 6 animals in each group) were used as biological replicates.

### 2.15. Western Blot

For Western blot analysis, brain tissue samples from 3 randomly selected animals per group (selected from the 6 animals in each group) were used, and protein concentrations were determined using a BCA protein assay kit. Each group included 3 samples, and protein concentrations were determined using a BCA protein assay kit. Equal amounts of protein were separated by 10% SDS-PAGE and transferred to PVDF membranes. The membranes were blocked with 5% non-fat dry milk for 2 h, then incubated overnight at 4 °C with the following primary antibodies: anti-MPO (1:3000), anti-PAD4 (1:3000), anti-CitH3 (1:1000), anti-TNF-α (1:1000), and anti-GAPDH (1:50,000). After washing with TBST buffer, the membranes were incubated with the corresponding secondary antibodies—goat anti-rabbit (1:20,000) and goat anti-mouse (1:10,000)—at room temperature for 2 h. Following another round of washing, the protein bands were visualized using a chemiluminescence imaging system (Bio-Rad Laboratories GmbH, Feldkirchen, Germany), and band intensities were quantified with ImageJ software.

### 2.16. Statistical Analysis

All statistical analyses were performed using GraphPad Prism software (version 10.1.2). Data are expressed as mean ± standard deviation (mean ± SD). The Shapiro–Wilk test was used to assess normality of data distribution, and homogeneity of variance was verified. For data meeting the assumptions of normality and homogeneity of variance, one-way analysis of variance (One-way ANOVA) was used for comparisons among multiple groups, followed by Dunnett’s post hoc test to specifically evaluate differences between each treatment group and the Model group. For data not meeting the assumption of normality, the Kruskal–Wallis non-parametric test was employed. A *p* value < 0.05 was considered statistically significant.

## 3. Results

### 3.1. Borneol Attenuated PMA-Induced NET Formation in Neutrophils

**Figure 1:**
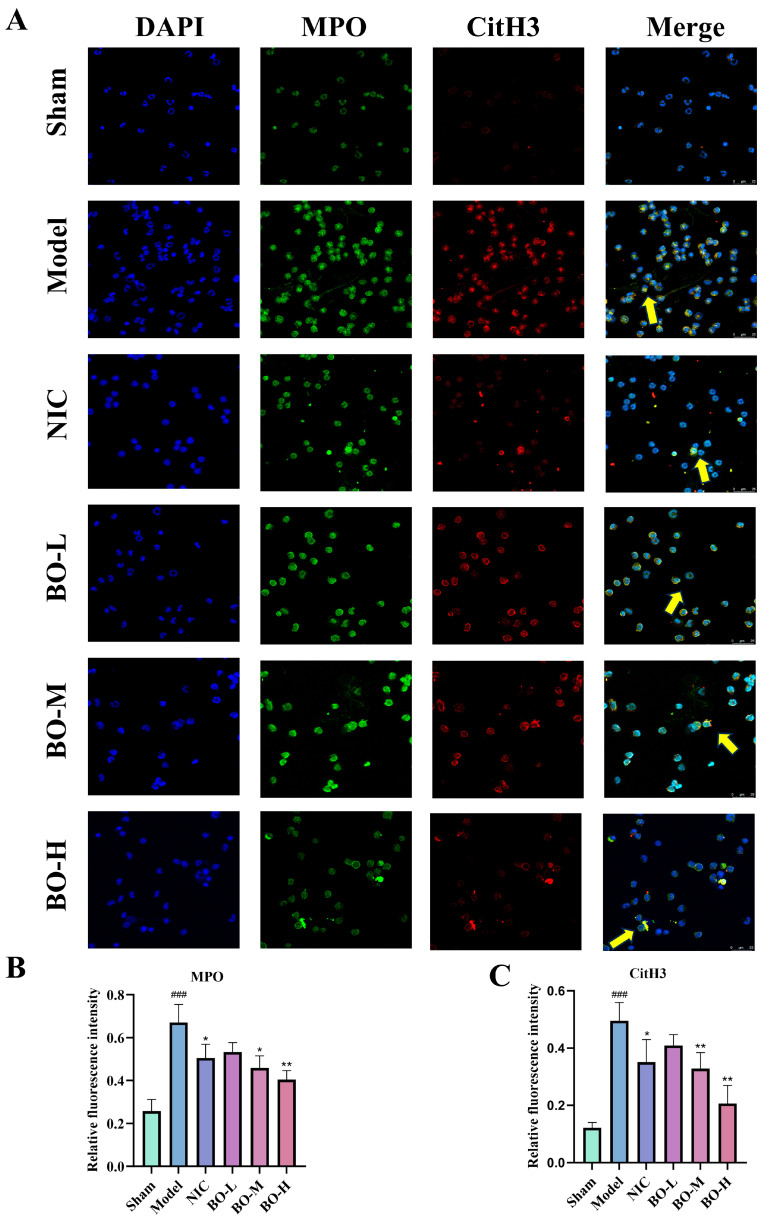
Borneol inhibits PMA-induced neutrophil extracellular trap (NET) formation in rat bone marrow-derived neutrophils: (**A**) Representative immunofluorescence staining images of neutrophils for DAPI, MPO, and CitH3. Blue indicates DAPI staining, green indicates MPO staining, and red indicates CitH3 staining. Yellow arrows indicate extracellular web-like NET-associated structures. Scale bar: 20 μm. (**B**,**C**) Quantification of MPO and CitH3 fluorescence intensities. Values are expressed as mean ± SD (*n* = 6). Data are analyzed by one-way ANOVA followed by Dunnett’s post hoc test. ### *p* < 0.001 versus the Sham group; * *p* < 0.05, ** *p* < 0.01, versus the Model group.

To evaluate the effect of borneol on NET formation in vitro, rat bone marrow-derived neutrophils were stimulated with PMA, and NETs were visualized by double immunofluorescence staining for MPO and CitH3. Representative images (Figure 1A) showed that the Sham group exhibited weak MPO and CitH3 fluorescence signals, with DAPI staining primarily localized within the nuclei and no obvious extracellular web-like structures. Following PMA stimulation, the Model group displayed markedly enhanced CitH3 and MPO fluorescence intensity, accompanied by chromatin decondensation and the formation of extracellular web-like structures, indicating increased NET formation. After borneol intervention, the fluorescence intensities of CitH3 and MPO were attenuated to varying degrees.

Quantitative analysis (Figure 1B,C) further revealed that the fluorescence intensities of MPO and CitH3 were significantly increased in the Model group compared with the Sham group (*p* < 0.001). Compared with the Model group, borneol at the medium and high doses significantly reduced both MPO and CitH3 fluorescence intensities (*p* < 0.05 or *p* < 0.01) and attenuated the extracellular web-like structures. The NIC group also showed a reduction in MPO and CitH3 fluorescence intensities (*p* < 0.05), although to a lesser extent than BO-H. These results suggest that BO-M and BO-H attenuate PMA-induced NET formation in neutrophils.

### 3.2. Borneol Improves Neurological Deficits and Hemodynamic Abnormalities

**Figure 2:**
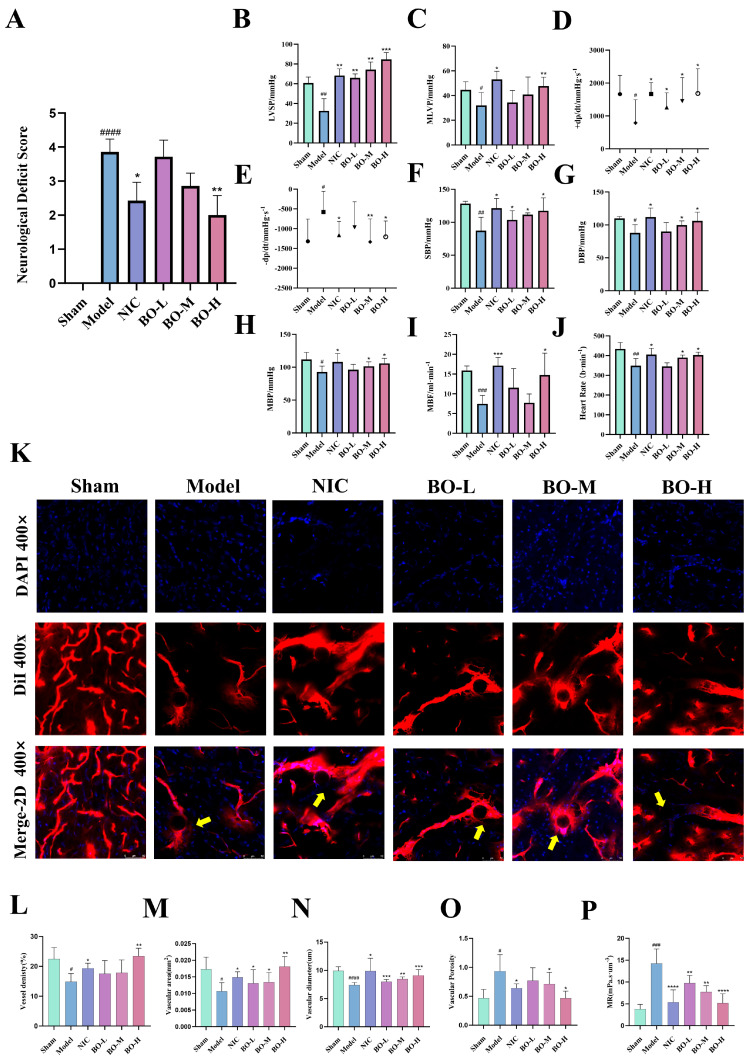
Borneol improves neurological function, hemodynamic parameters, and cerebral microvascular perfusion in rats with microcirculatory dysfunction-associated brain injury: (**A**) Longa score. (**B**–**J**) Hemodynamic parameters. (**K**) Representative images of DiI-labeled cerebral microvessels. Blue indicates DAPI-stained nuclei, and red indicates DiI-labeled cerebral microvessels. Red tubular structures represent cerebral microvessels. Yellow arrows indicate microsphere retention and impaired perfusion. Scale bar: 50 μm. (**L**–**P**) Quantitative analysis of average vascular diameter, vascular area, vessel density, vascular porosity, and microvascular resistance (MR). Data are presented as mean ± SD or median (interquartile range). *n* = *6*. Data are analyzed by one-way ANOVA followed by Dunnett’s post hoc test for normally distributed data, or Kruskal–Wallis test for non-normally distributed data. # *p* < 0.05, ## *p* < 0.01, ### *p* < 0.001, and #### *p* < 0.0001 versus the Sham group; * *p* < 0.05, ** *p* < 0.01, *** *p* < 0.001, and **** *p* < 0.0001 versus the Model group. For AngioTool-based microvascular analyses, data from multiple fields per animal are averaged, with the animal as the statistical unit (*n* = 6 per group).

To evaluate the neuroprotective effects of borneol on brain injury in rats with microcirculatory dysfunction, we used the Longa scoring system to evaluate neurological deficits. The results showed (Figure 2A) that compared with the Sham group, the Model group exhibited significantly higher neurological function deficit scores (*p* < 0.0001), whereas the BO-H group significantly reduced the neurological function deficit scores (*p* < 0.01), indicating that BO may facilitate neurological recovery after brain injury. The NIC group exhibited significantly reduced neurological deficit scores (*p* < 0.05).

Hemodynamic measurements showed (Figure 2B–J) that compared with the Sham group, the Model group exhibited significant deterioration in indicators such as left ventricular systolic pressure (LVSP), left ventricular mean pressure (MLVP), the maximum rate of increase in left ventricular pressure (+dp/dt), the maximum rate of decrease in left ventricular pressure (−dp/dt), arterial systolic pressure (SBP), arterial diastolic pressure (DBP), mean blood pressure (MBP), mean blood flow (MBF), and heart rate (HR). Compared with the Model group, the BO-L group significantly improved LVSP, dp/dt, and SBP; the BO-M group showed noticeable improvements in LVSP, ±dp/dt, SBP, DBP, MBP, and HR; Both the high-dose borneol (BO-H) and NIC positive control groups yielded significant improvements in LVSP, ±dp/dt, MLVP, SBP, DBP, MBP, MBF, and HR (*p* < 0.05). This observation aligns with nicorandil’s well-established vasodilatory and KATP channel-opening activities. Medium- and high-dose borneol also produced favorable alterations in hemodynamic indicators, indicating that borneol exerts protective effects on hemodynamic function.

### 3.3. Borneol Improved Cerebral Microvascular Perfusion and Reduced Microvascular Resistance

DiI perfusion imaging results showed (Figure 2K) that the cortical microvascular network structure of the Sham group rats was intact and continuous, with clear vascular contours and uniform perfusion distribution; whereas the Model group displayed a large number of microspheres retained in the microvascular lumen, accompanied by vasoconstriction, local perfusion interruption, and sparse microvascular networks, indicating that cerebral microvascular perfusion was significantly impaired following microsphere embolization.

Further quantitative analysis results (Figure 2L–P) showed that compared with the Sham group, the Model group had significantly reduced average vascular diameter, vascular area, and vessel density (*p* < 0.05 or *p* < 0.0001), while vascular porosity was significantly increased (*p* < 0.05); estimates of microvascular resistance (MR) based on the Poiseuille formula indicated a significant increase in microvascular resistance in the Model group (*p* < 0.001), demonstrating obvious cerebral microcirculatory dysfunction in the model animals. Compared with the Model group, the BO-L group significantly improved average vascular diameter, vascular area, and microvascular resistance; the BO-M group further significantly improved vascular porosity on this basis; Both the BO-H group and NIC group significantly elevated vascular diameter, vascular area and vessel density, while markedly decreasing vascular porosity and microvascular resistance (*p* < 0.05). These results indicate that borneol can effectively improve microsphere-induced cerebral microvascular perfusion disorders and alleviate impaired microcirculation flow.

### 3.4. Borneol Alleviated Hemorheological Abnormalities and Improved Hematological Indices

**Figure 3:**
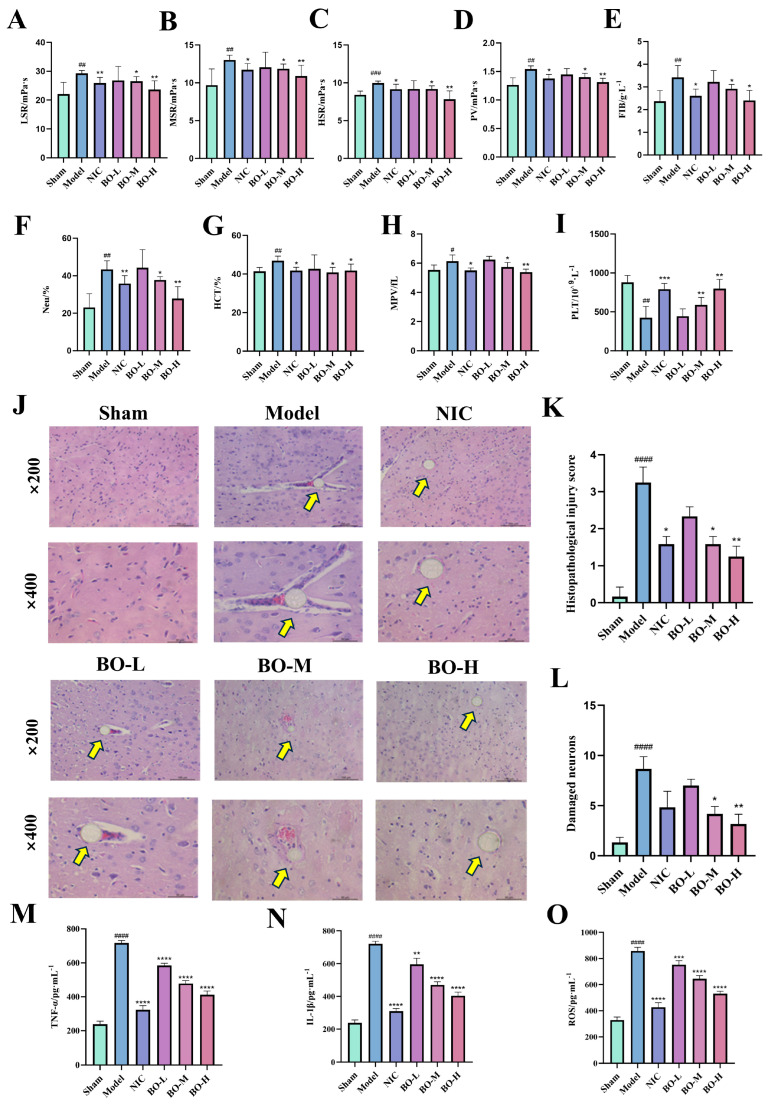
Borneol improves hemorheological abnormalities and brain histopathological injury in rats with microcirculatory dysfunction-associated brain injury: (**A**–**I**) Analysis of hemorheological parameters and related indices. (**J**) Representative hematoxylin and eosin (HE) staining images of brain tissue. Yellow arrows indicate retained polyethylene microspheres, erythrocyte extravasation, and local tissue injury. (**K**,**L**) Quantitative analysis of histopathological injury, including H&E histopathological injury score (0–4) and damaged neurons per high-power field. (**M**–**O**) Serum levels determined by ELISA. Data are presented as mean ± SD (*n* = 6). Data in panels (**A**–**I**,**M**–**O**) are analyzed by one-way ANOVA followed by Dunnett’s post hoc test. Histopathological data in panels (**K**,**L**) are analyzed using the Kruskal–Wallis test followed by Dunn’s multiple comparisons test. # *p* < 0.05, ## *p* < 0.01, ### *p* < 0.001, and #### *p* < 0.0001 versus the Sham group; * *p* < 0.05, ** *p* < 0.01, *** *p* < 0.001, and **** *p* < 0.0001 versus the Model group.

To further evaluate the effect of borneol on hemorheology and hematological parameters, this study measured whole blood viscosity, plasma viscosity (PV), fibrinogen (FIB), and main hematological parameters. As shown in Figure 3A–I, compared with the Sham group, the Model group had significantly increased whole blood viscosity at low (LSR), medium (MSR), and high shear rates (HSR) (*p* < 0.01 or *p* < 0.001), while PV and FIB levels were also significantly elevated (*p* < 0.01), suggesting a hyperviscous and prothrombotic state in model rats; in addition, the proportions of neutrophils (Neu), hematocrit (HCT), and mean platelet volume (MPV) in the Model group were significantly increased (*p* < 0.05 or *p* < 0.01), while platelet count (PLT) was significantly decreased (*p* < 0.01). NIC group significantly reduced WBV at all shear rates, PV, FIB, Neu, HCT, and MPV, and increased PLT (*p* < 0.05), consistent with its known vasodilatory and microcirculation-improving properties. After borneol intervention, the BO-L group showed no significant improvement in the above abnormalities; however, the BO-M and BO-H dose groups had significantly decreased LSR, MSR and HSR, as well as PV and FIB (*p* < 0.05 or *p* < 0.01), while Neu, HCT, and MPV were significantly reduced (*p* < 0.05 or *p* < 0.01), and PLT was significantly increased (*p* < 0.01). These results indicate that BO can alleviate pathological hemorheological abnormalities and improve abnormal changes in hematological parameters.

### 3.5. Borneol Attenuated Histopathological Brain Injury

HE staining showed (Figure 3J) that in the Sham group, the cortical and hippocampal structures of rats were essentially intact, neurons were arranged regularly, nuclear structures were clear, and no significant pathological changes were observed. In contrast, the brain tissues in the Model group exhibited obvious pathological damage, including retention of microbead fragments in microvessels or adjacent brain parenchyma, local extravasation of red blood cells, as well as some neurons appearing shrunken, with increased cytoplasmic eosinophilia, vacuolar changes, widened intercellular spaces, and nuclear pyknosis, accompanied by a certain degree of inflammatory cell infiltration, indicating significant pathological damage following microcirculatory disturbance. Compared with the Model group, the pathological changes were alleviated to varying degrees after borneol intervention, manifested as milder tissue damage around the microbeads, reduced local red blood cell extravasation and inflammatory cell infiltration, and partial improvement in neuronal morphology. The NIC group also showed alleviated pathological damage, with reduced erythrocyte extravasation and inflammatory infiltration. These results suggest that borneol has a certain mitigating effect on brain tissue pathological damage caused by microcirculatory dysfunction.

Semi-quantitative analysis further supported the H&E observations (Figure 3K,L). The Model group showed a significantly higher histopathological injury score and more damaged neurons per high-power field than the Sham group. Compared with the Model group, NIC and borneol treatment reduced the histopathological injury score and damaged neuron counts to varying degrees, with the most evident reduction observed in the BO-H group. These quantitative results further support that borneol attenuated histopathological injury in polyethylene microsphere-induced cerebral microcirculatory dysfunction.

### 3.6. Borneol Reduces Inflammatory Response and Oxidative Stress Levels

To evaluate the effect of borneol on serum inflammatory response and oxidative stress in a rat model of microcirculatory dysfunction, this study used ELISA to measure serum TNF-α, IL-1β and ROS levels. The results showed (Figure 3M–O) that, compared with the Sham group, the Model group had significantly increased serum levels of TNF-α, IL-1β and ROS (all *p* < 0.0001), indicating that brain injury induced by microcirculatory dysfunction was accompanied by marked inflammatory responses and oxidative stress. Compared with the Model group, BO-L, BO-M and BO-H significantly reduced serum TNF-α, IL-1β and ROS levels (*p* < 0.01). The NIC group significantly reduced serum TNF-α and IL-1β levels (*p* < 0.0001), consistent with its anti-inflammatory effects under microcirculatory dysfunction conditions. The results indicate that borneol can alleviate inflammatory response and oxidative stress in rats with microcirculatory dysfunction.

### 3.7. Borneol Reduced NET-Related Signals in Brain Tissue

**Figure 4:**
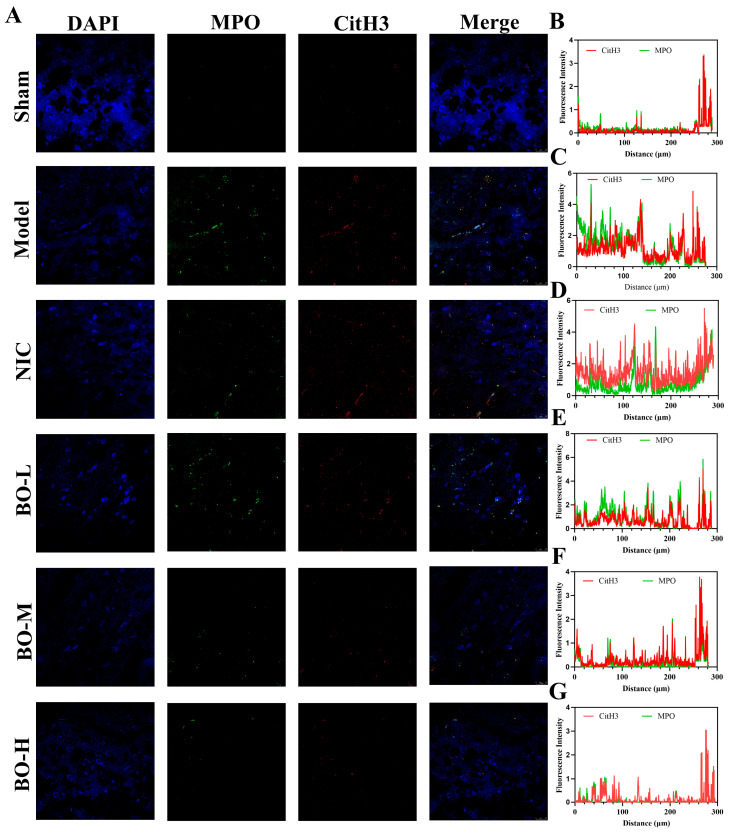
Borneol reduces MPO/CitH3-positive NET-related signals in brain tissue: (**A**) Representative immunofluorescence images of DAPI, MPO, CitH3, and merged staining in brain tissue. Blue indicates DAPI staining, green indicates MPO staining, and red indicates CitH3 staining. MPO/CitH3-positive signals are interpreted as NET-related signals rather than definitive extracellular NET structures. (**B**–**G**) Representative line-scan fluorescence intensity profiles showing the spatial distribution of MPO and CitH3 signals in the Sham, Model, NIC, BO-L, BO-M, and BO-H groups, respectively. Scale bar: 25 μm.

Given that the in vitro experiments showed that borneol inhibited PMA-induced NET formation in neutrophils, this study further examined NET-related marker signals in brain tissue. Representative immunofluorescence images showed weak MPO and CitH3 signals in the Sham group, whereas the Model group exhibited increased MPO/CitH3-positive signals in brain tissue (Figure 4A). Representative line-scan fluorescence intensity profiles showed partial spatial overlap between MPO and CitH3 signals in the Model group, which was consistent with increased MPO/CitH3-positive NET-related signals. Compared with the Model group, NIC and borneol treatment, particularly BO-M and BO-H, visibly attenuated these signals and reduced their spatial overlap (Figure 4B–G). Because MPO/CitH3 immunofluorescence alone cannot definitively identify extracellular NET structures, these findings were interpreted as changes in NET-related marker signals rather than direct evidence of definitive NET structures.

### 3.8. Borneol Downregulated the Expression of NET- and Inflammation-Related Molecules

**Figure 5:**
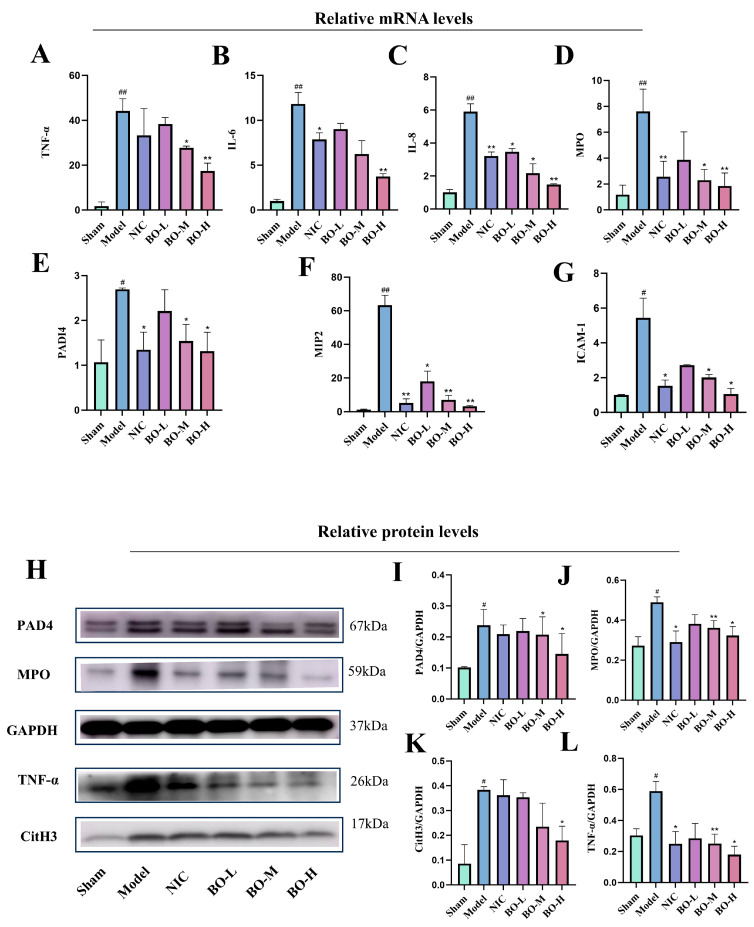
Borneol downregulates NET-related and inflammatory molecules in brain tissue of rats with microcirculatory dysfunction-associated brain injury: (**A**–**G**) Relative mRNA levels of TNF-α, IL-6, IL-8, MPO, PADI4, MIP2, and ICAM-1 determined by quantitative real-time PCR. (**H**) Representative Western blot bands of PAD4, MPO, TNF-α, CitH3, and GAPDH. (**I**–**L**) Quantitative analysis of relative protein levels of PAD4, MPO, CitH3, and TNF-α normalized to GAPDH. Data are presented as mean ± SD (*n* = 3). Data are analyzed by one-way ANOVA followed by Dunnett’s post hoc test. # *p* < 0.05, ## *p* < 0.01, versus the Sham group; * *p* < 0.05, ** *p* < 0.01, versus the Model group.

At the transcriptional level, qPCR results showed (Figure 5A–G) that compared with the Sham group, the Model group had significantly elevated mRNA expression of TNF-α, IL-6, IL-8, MPO, *PADI4*, MIP2, and ICAM-1 in the brain tissue (*p* < 0.05); compared with the Model group, the BO-L group significantly reduced IL-8 and MIP2 mRNA levels (*p* < 0.05), the BO-M group significantly reduced mRNA expression of IL-6, IL-8, MPO, *PADI4*, MIP2, and ICAM-1 (*p* < 0.05 or *p* < 0.01), and the BO-H group significantly reduced mRNA expression of TNF-α, IL-6, IL-8, MPO, *PADI4*, MIP2, and ICAM-1 (*p* < 0.05 or *p* < 0.01). The NIC group significantly reduced mRNA expression of IL-6, IL-8, MPO, *PADI4*, MIP2, and ICAM-1 (*p* < 0.05 or *p* < 0.01). These results indicate that borneol can inhibit abnormal expression of inflammation- and NET-related genes in brain tissue.

Western blot results showed (Figure 5H–L) that in the Model group, protein expression of PAD4, MPO, CitH3, and TNF-α in brain tissue was significantly increased compared with the Sham group (*p* < 0.05), suggesting abnormal upregulation of NET-related molecules and inflammatory factors in the brain after microcirculatory dysfunction. Compared with the Model group, the BO-L group showed no significant downregulation; the BO-M group significantly reduced PAD4, MPO, and TNF-α protein expression in brain tissue (*p* < 0.05 or *p* < 0.01); the BO-H group significantly downregulated PAD4, MPO, CitH3, and TNF-α protein expression in brain tissue (*p* < 0.05). The NIC group significantly downregulated MPO and TNF-α protein expression in brain tissue (*p* < 0.05). These results indicate that borneol treatment was associated with reduced NET-related marker expression and decreased inflammation-related molecules in brain tissue after microcirculatory dysfunction.

## 4. Discussion

Microcirculatory dysfunction is characterized by microvascular occlusion, inadequate tissue perfusion, hemorheological abnormalities, and increased microvascular resistance. These alterations are relevant to microcirculation-related brain injury and impaired microvascular perfusion [10,19,20]. Therefore, experimental models that isolate cerebral microcirculatory obstruction may provide valuable mechanistic insights into the failure of microvascular perfusion.

In this study, a rat model of polyethylene microsphere-induced cerebral microcirculatory dysfunction was established to replicate mechanical microvascular obstruction, hypoperfusion, increased microvascular resistance, and secondary brain tissue injury. The model rats exhibited significant neurological deficits, hemodynamic disorders, impaired microvascular perfusion, abnormal hemorheology, and pathological damage to brain tissue. Notably, these functional and structural alterations were accompanied by elevated NET-related markers (MPO, CitH3, PAD4) and pro-inflammatory cytokines (TNF-α, IL-1β, IL-6) in both brain tissue and serum. These findings suggest that microcirculatory dysfunction in this model is closely associated with increased NET-related markers, which may contribute to the observed neurological deficits. Borneol treatment, particularly at medium and high doses, significantly improved neurological scores, reduced NET-related markers, and restored microvascular perfusion. These improvements occurred in parallel with reductions in NET-related markers, although a causal link between these events remains to be established.

The relationship between neurological deficits and microcirculatory dysfunction in this model is likely mediated through multiple interconnected pathways. Microvascular obstruction caused by polyethylene microspheres may lead to regional hypoperfusion and tissue hypoxia [4,19], which could be associated with endothelial activation, neutrophil recruitment, and increased NET-related marker signals. These NET-related changes may further contribute to microvascular inflammation and perfusion impairment [8,10,13,21]. Borneol may mitigate this process through improvements in hemodynamics and microvascular perfusion, together with reductions in oxidative stress, inflammatory responses, and NET-related markers. However, the Longa scale used in this study, while widely accepted [17], is a coarse 0–4 ordinal scale with limited sensitivity for detecting subtle functional improvements; lower doses exhibited improving trends that did not achieve statistical significance. Importantly, dose-dependent improvements were consistently observed across multiple independent quantitative readouts directly linked to neurovascular integrity, including enhanced microvascular perfusion, attenuated histopathological damage, and downregulated NET-related and inflammatory mediators, providing supportive evidence for the functional improvements suggested by the Longa score. We acknowledge that more sensitive behavioral assays, such as the Rotarod test, corner test, or adhesive removal test, would yield more comprehensive functional assessments [22,23]. However, due to the severity of acute microsphere-induced injury, the animals could not reliably perform the Rotarod test within the 24 h observation window, raising ethical concerns; therefore, this test was not conducted. Future studies will incorporate more sensitive behavioral test batteries with extended observation periods.

It should be noted that borneol improved multiple systemic hemodynamic parameters, including blood pressure, carotid blood flow, heart rate, and ventricular pressure indices. These cardiovascular effects could independently enhance cerebral perfusion, thereby secondarily reducing hypoxia, endothelial activation, neutrophil recruitment, and inflammation. Thus, the observed decreases in MPO, CitH3, PAD4, and inflammatory cytokines may partially reflect improved hemodynamic and microcirculatory status rather than a primary NET-specific action of borneol. However, as we did not perform mediation or adjusted analyses to separate these direct and indirect effects, this alternative explanation remains a key limitation. Future studies employing statistical adjustment or pathway-specific inhibitors are warranted to clarify whether borneol’s protective effects are primarily attributable to its systemic hemodynamic actions or to more specific modulation of NET-related pathways.

Nicorandil was selected as the positive control for this study due to its dual-function properties, which include nitrate-like nitric oxide (NO)-donating activity and the ability to open ATP-sensitive potassium (KATP) channels. It has been reported to improve microvascular perfusion and provide protective effects in conditions associated with ischemic or microcirculatory dysfunction [24,25]. Previous research has also employed nicorandil in models related to microembolization or no-reflow, thereby affirming its relevance as a microcirculation-oriented positive control [26]. Notably, given that the primary objective of this study was to evaluate the effects of borneol on microcirculatory dysfunction and perfusion parameters, nicorandil was selected specifically as a hemodynamic and microcirculatory comparator rather than a NET-specific positive control. The reductions in NET-related markers observed with nicorandil treatment are considered secondary to its hemodynamic improvement, which serves as a reference for interpreting borneol’s effects. For future mechanistic validation of NET-specific actions, we recognize that DNase I or PAD4 inhibitors would be more appropriate comparators, and such investigations are planned.

Recent studies have demonstrated that neutrophil extracellular traps (NETs) play a crucial role in microcirculatory dysfunction [8,13]. NETs serve as an adhesive scaffold for platelets, red blood cells, and coagulation factors through their DNA backbone and granular proteins, thereby promoting the formation of microthrombosis [12,27,28]. Additionally, they can damage vascular endothelium, amplify local inflammatory responses, and further exacerbate microvascular obstruction and perfusion impairment [29]. Our observations indicate that rats in the Model group exhibited significant impairment of cerebral microvascular perfusion, elevated microvascular resistance, and abnormal hemodynamics, which were accompanied by a synchronized upregulation of NET-related markers, including MPO, CitH3, and PAD4, in brain tissue. These findings suggest a pathological association among microcirculatory impairment, inflammation, and NETosis.

The microvascular resistance values estimated via the Hagen–Poiseuille equation in this study should be interpreted as relative indicators rather than absolute physiological parameters, as the equation was applied to simplified geometric parameters derived from 2D fluorescence images and could not fully account for the complexity of the cerebral microvascular network, including branching, tortuosity, and regional variations in perfusion pressure. Subsequent studies in our laboratory will incorporate three-dimensional vascular reconstruction and pressure-flow measurements to provide more accurate assessments.

Borneol (BO), a traditional aromatic resuscitative medicine, has been utilized in clinical practice for the treatment of acute and critical conditions, including stroke-related coma, for an extended period. Various borneol preparations, as listed in the 2020 edition of the Chinese Pharmacopoeia, have been widely applied in the management of cardiovascular and cerebrovascular diseases. For instance, the borneol-containing Xingnaojing injection is frequently employed to enhance consciousness and improve microcirculation in stroke patients [30]. Additionally, compound Danshen dripping pills underscore the clinical significance of borneol-like components in brain protection and microcirculation enhancement. Furthermore, edaravone dexborneol is a clinically recognized neuroprotective agent for acute ischemic stroke, with recent trials and reviews indicating its therapeutic efficacy in brain protection [31,32,33]. These clinical and pharmacological findings suggest that borneol may exert immunopharmacological effects by improving microcirculation, suppressing inflammatory responses, and safeguarding the blood–brain barrier [34]. However, the potential of borneol to modulate NET-associated inflammatory responses in the context of cerebral microcirculatory dysfunction remains unclear. Given its documented anti-inflammatory, antioxidant, and microcirculation-improving properties, we hypothesize that the protective effects of borneol in microcirculatory dysfunction-associated brain injury may coincide with reduced NET-related pathological activation, rather than being directly mediated by it.

NET formation is primarily driven by oxidative stress-dependent activation of PAD4, histone citrullination, chromatin decondensation, and the extrusion of DNA-associated granular proteins [35,36]. Under PMA stimulation or ischemic conditions, the activation of NADPH oxidase in neutrophils induces a substantial production of reactive oxygen species (ROS), which subsequently promotes PAD4-mediated histone H3 citrullination, ultimately leading to chromatin decondensation and the release of NETs [13,37]. Therefore, the ROS/PAD4/CitH3 pathway is recognized as a crucial molecular basis for NET formation [35]. In our study, the model group exhibited concurrent increases in ROS, PAD4, CitH3, and myeloperoxidase (MPO), whereas treatment with borneol mitigated these alterations. Although these findings do not establish a direct causal interaction between borneol and the ROS/PAD4/CitH3 axis, they demonstrate a consistent association between borneol treatment and reduced NET-related markers. Whether this association reflects a direct functional inhibition of NETosis, or is secondary to improved hemodynamics and reduced systemic inflammation, cannot be determined from the current data. This interpretation aligns with previous reports detailing the antioxidant and anti-inflammatory properties of borneol in the context of cerebral ischemic injury and microcirculatory dysfunction. Therefore, this study identifies NET-related inflammation as a correlative phenotype, and further research is required to establish causal links between borneol’s established pharmacological activities and its protective impacts on cerebral microcirculation.

This study demonstrated that borneol exerts dose-dependent protective effects against microcirculatory dysfunction-associated brain injury, potentially linked to the reduction in NET-related inflammatory responses. Previous research has primarily concentrated on the anti-inflammatory, antioxidant, and blood–brain barrier-protective effects of borneol. In contrast, the present study investigated the protective effects of borneol against microcirculatory dysfunction-associated brain injury from the perspective of NET-related inflammatory regulation. By integrating an in vitro NETs model with an in vivo model of microcirculatory dysfunction, we obtained mutually supportive evidence across cellular, tissue, and whole-animal functional levels. These findings provide a new mechanistic explanation for the protective effects of borneol, support the pharmacological basis of existing borneol-containing preparations for brain protection, and offer experimental evidence for the development of novel therapeutic strategies for microcirculatory dysfunction-related brain injury.

Several limitations should be acknowledged. First, borneol possesses multiple pharmacological effects. The reduction in NET-related markers observed in this study may result from the direct modulation of NETosis by borneol, or secondary improvements in microcirculatory perfusion and inflammatory status. Further mechanistic validation, including DNase I degradation assay, PAD4 gene knockout, neutrophil depletion, and NET rescue experiments, is required to confirm the direct causal relationship between borneol-mediated NET regulation and cerebral protection. Second, the polyethylene microsphere model induces permanent mechanical microvascular occlusion with inert particles and does not fully reproduce thrombotic occlusion, fibrinolysis, reperfusion injury, or ischemic core–penumbra evolution in clinical stroke [4]. Although microcirculatory dysfunction and no-reflow-like perfusion failure are clinically relevant after macrovascular recanalization [19,20,38], the present model should be regarded as a simplified model of cerebral microvascular obstruction. Third, functional assessment relied mainly on the Longa score; future studies should include more sensitive assays, such as the Rotarod, corner, and adhesive removal tests, with longer follow-up. Finally, systemic hemodynamic effects, the prophylactic dosing regimen (borneol was given before microsphere injection, the exploratory high dose of borneol, the 24 h observation window, the exclusive use of male rats without exploring sex differences, and the non-definitive nature of MPO/CitH3-based NET detection should be considered when interpreting these findings. Overall, this study provides preliminary experimental evidence suggesting that NET-related inflammatory activation may contribute to microcirculatory dysfunction-associated brain injury and may be modulated by borneol.

## 5. Conclusions

This study investigated the protective effects and associated NET-related changes in borneol against brain injury associated with microcirculatory dysfunction. Borneol inhibited PMA-induced NET formation in neutrophils in vitro, and in vivo improved neurological function, cerebral microvascular perfusion, hemodynamics, and histopathological damage in rats with microcirculatory dysfunction-associated brain injury. In addition, borneol reduced inflammatory responses and oxidative stress, and downregulated the expression of NET- and inflammation-related molecules in brain tissue. These findings suggest that borneol alleviates microcirculatory dysfunction-associated brain injury, with reductions in NET-related markers and improved microvascular perfusion representing exploratory and associative observations. Nevertheless, given the prophylactic administration schedule used in this study, further post-injury treatment studies are needed to assess its translational potential.

## Figures and Tables

**Table 1:** The sequences of primers.

Gene	Forward Primer (5′-3′)	Reverse Primer (5′-3′)
MPO	ACACCCTCATCCAACCCTT	ACCTTCCAGCACAACTCTCC
*PADI4*	CTTTCGGCTGCTACTGTCC	TGATTGTCTGCCTTTTCCTCT
TNF-α	TGTGCCTCAGCCTCTTCTC	AACTTCTCCTCCTTGTTGGG
IL-6	TCTTGGGACTGATGTTGTTG	TAAGCCTCCGACTTGTGAA
IL-8	GGACAGAACATAAGCCAACA	CTTTCATCACACAGGACAGG
ICAM-1	TATCCATCCATCCCACAGAA	CATCCAGTTAGTCTCCAACCC
MIP2	AATGCCTGACGACCCTACC	TCAGTTAGCCTTGCCTTTGTTC
β-actin	CACCCGCGAGTACAACCTTC	CCCATACCCACCATCACACC

## Data Availability

The data presented in this study are available upon request from the corresponding author due to ethical restrictions related to the animal experimental records.
